# The epidemiology of tick-borne haemoparasites as determined by the reverse line blot hybridization assay in an intensively studied cohort of calves in western Kenya

**DOI:** 10.1016/j.vetpar.2015.02.020

**Published:** 2015-05-30

**Authors:** Nyawira E. Njiiri, B. Mark deC. Bronsvoort, Nicola E. Collins, Helena C. Steyn, Milana Troskie, Ilse Vorster, S.M. Thumbi, Kgomotso P. Sibeko, Amy Jennings, Ilana Conradie van Wyk, Mary Mbole-Kariuki, Henry Kiara, E. Jane Poole, Olivier Hanotte, Koos Coetzer, Marinda C. Oosthuizen, Mark Woolhouse, Philip Toye

**Affiliations:** a The International Livestock Research Institute, PO Box, 30709, 00100 Nairobi, Kenya; b Department of Veterinary Tropical Diseases, Faculty of Veterinary Science, University of Pretoria, Private Bag X04, Onderstepoort 0110, South Africa; c The Roslin Institute, University of Edinburgh, Easter Bush, Edinburgh EH25 9RG, UK; d Agricultural Research Council-Onderstepoort Veterinary Institute, Private Bag X05, Onderstepoort 0110, South Africa; e Centre for Immunity, Infection & Evolution, University of Edinburgh, Edinburgh EH9 3JT, UK; f School of Life Sciences, University of Nottingham, Nottingham NG7 2RD, UK; g Paul G. Allen School for Global Animal Health, Washington State University, Pullman, WA 99164-7090, USA; h The Royal (Dick) School of Veterinary Studies, University of Edinburgh, Easter Bush, Edinburgh EH25 9RG, UK; i African Union InterAfrican Bureau for Animal Resources (AU-IBAR), PO Box 30786, 00100 Nairobi, Kenya

**Keywords:** *Theileria*, *Anaplasma*, Haemoparasites, Reverse line blot, Coinfection, Cattle

## Abstract

The development of sensitive surveillance technologies using PCR-based detection of microbial DNA, such as the reverse line blot assay, can facilitate the gathering of epidemiological information on tick-borne diseases, which continue to hamper the productivity of livestock in many parts of Africa and elsewhere. We have employed a reverse line blot assay to detect the prevalence of tick-borne parasites in an intensively studied cohort of indigenous calves in western Kenya. The calves were recruited close to birth and monitored for the presence of infectious disease for up to 51 weeks. The final visit samples from 453 calves which survived for the study period were analyzed by RLB. The results indicated high prevalences of *Theileria mutans* (71.6%), *T. velifera* (62.8%), *Anaplasma* sp*.* Omatjenne (42.7%), *A. bovis* (39.9%), *Theileria* sp*.* (sable) (32.7%), *T. parva* (12.9%) and *T. taurotragi* (8.5%), with minor occurrences of eight other haemoparasites. The unexpectedly low prevalence of the pathogenic species *Ehrlichia ruminantium* was confirmed by a species-specific PCR targeting the pCS20 gene region. Coinfection analyses of the seven most prevalent haemoparasites indicated that they were present as coinfections in over 90% of the cases. The analyses revealed significant associations between several of the *Theileria* parasites, in particular *T. velifera* with *Theileria* sp. sable and *T. mutans*, and *T. parva* with *T. taurotragi.* There was very little coinfection of the two most common *Anaplasma* species, although they were commonly detected as coinfections with the *Theileria* parasites. The comparison of reverse line blot and serological results for four haemoparasites (*T. parva*, *T. mutans*, *A. marginale* and *B. bigemina*) indicated that, except for the mostly benign *T. mutans*, indigenous cattle seem capable of clearing infections of the three other, pathogenic parasites to below detectable levels*.* Although the study site was located across four agroecological zones, there was little restriction of the parasites to particular zones.

## Highlights

• A reverse line blot assay was used to estimate tick-borne haemoparasite prevalence in an intensively studied cohort of indigenous cattle in western Kenya.
• There were high prevalences of *Theileria mutans* (71.6%), *T. velifera* (62.8%), *Anaplasma* sp*.* Omatjenne (42.7%), *A. bovis* (39.9%), *Theileria* sp*.* (sable) (32.7%), *T. parva* (12.9%) and *T. taurotragi* (8.5%), with minor occurrences of eight other haemoparasites.
• The most prevalent haemoparasites were mostly present as coinfections, with strong associations between several of the *Theileria* parasites, in particular *T. velifera* with *Theileria* sp. sable and *T. mutans*, and *T. parva* with *T. taurotragi*.
• Comparison of RLB and serological results indicated that indigenous cattle seem capable of clearing infections of three pathogenic parasites (*T. parva*, *A. marginale* and *B. bigemina*), whereas infections with the mostly benign *T. mutans* are more persistent.

## 1 Introduction

Tick-borne infections in cattle in Africa are complex, with many tick species interacting with different hosts and transmitting a wide range of pathogenic and non-pathogenic organisms. The diseases caused by tick-borne pathogens cause substantial economic loss (Uilenberg, 1995) and the improvement of strategies to control the diseases caused by these organisms requires more detailed knowledge of their prevalence and how they interact with each other. In Kenya, the most important tick-borne diseases are theileriosis, anaplasmosis, babesiosis and heartwater (Wesonga et al., 2010), with East Coast fever (ECF), caused by the protozoan parasite *Theileria parva*, being of particular importance. ECF causes substantial production losses through mortality and decreased productivity (Mukhebi et al., 1992), and is a major constraint to keeping improved breeds of cattle in endemic areas (Conelly, 1998; Gitau et al., 2001). Currently, the only practical means of controlling the disease are regular application of acaricides or immunization using the infection and treatment method (Di Giulio et al., 2009).

The application of laboratory diagnostic assays to determine the prevalence of infections is well established. Microscopy is commonly used for diagnosis of tick-borne diseases, because it is both easy to carry out and cheap. However, the method is relatively insensitive and the organisms are difficult to find and identify to the level of species. Serological methods including indirect fluorescent antibody tests (IFAT) and enzyme-linked immunosorbent assays (ELISA) are used to detect antibodies in animals that have been exposed to infections, but these do not necessarily reveal current infections. Several PCR diagnostic techniques have been developed to detect single parasite species, such as the pCS20 quantitative real-time PCR (qPCR) assay for the detection of *Ehrlichia ruminantium* (Steyn et al., 2008) and the nested p104 PCR assay for the detection of *T. parva* (Skilton et al., 2002). A reverse line blot (RLB) hybridization assay has been developed to detect and differentiate between several parasite species simultaneously. The initial RLB assay was developed to detect all the *Theileria* and *Babesia* species that infect cattle (Gubbels et al., 1999). Bekker et al. (2002) described a further development of this assay that enabled simultaneous detection of all the *Anaplasma* and *Ehrlichia* species that infect ruminants. In eastern Africa, a combination of these techniques has been applied to field samples for the identification of tick-borne haemoparasites in an endemic region in Uganda (Oura et al., 2004). In their study, the RLB assay was assessed for the ability to detect the principal tick-transmitted protozoan and rickettsial cattle pathogens in indigenous and crossbred cattle and to identify the carrier states of the parasites. The assay was able to identify *T. parva* at a level comparable with previously developed PCR methods and well below conventional microscopic detection. More recently, Asiimwe et al. (2013) also used the RLB assay to determine the prevalence of haemoparasites and their infection kinetics in cattle on a single farm in central Uganda.

The aim of the study reported here was to gain further information on the prevalence of tick-borne parasites in an important farming system in eastern Africa through the use of the RLB assay. The samples were obtained from an intensively studied birth cohort of 548 calves in western Kenya (Bronsvoort et al., 2013). Apart from baseline prevalence, the results were used to determine the levels of coinfections in the calf population.

## 2 Materials and methods

### 2.1 Animals

**Fig. 1:**
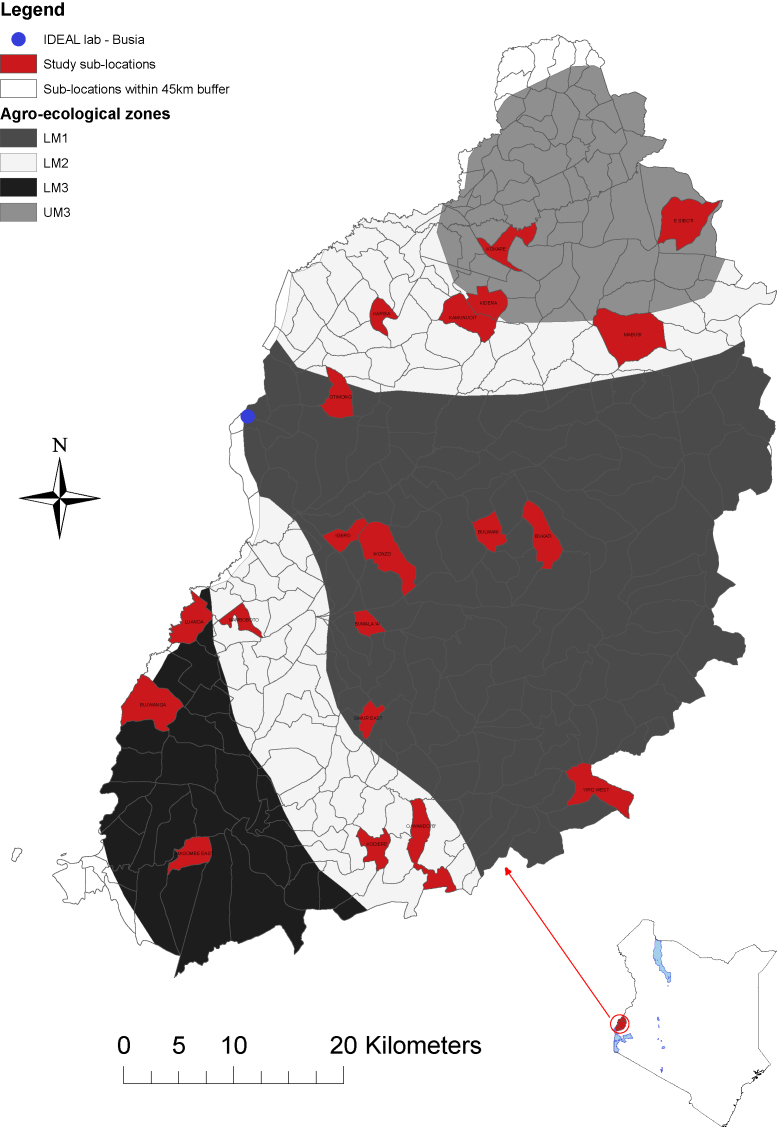
Map of western Kenya showing the study site and AEZs (Bronsvoort et al., 2013).

The IDEAL project has been described in detail elsewhere (Bronsvoort et al., 2013). In brief, a cohort of 548 East African shorthorn calves was recruited into the project at birth and monitored for a period of one year. The calves were visited every 5 weeks, at which time they were subject to a complete physical examination and samples, including blood, were taken for further analysis. The calves were chosen from 20 randomly selected sub-locations in western Kenya, which were distributed across four agro-ecological zones (AEZs). The AEZs are defined according to climate, altitude and agricultural activities (Jaetzold and Schmidt, 1983). As shown in Fig. 1, the study region encompassed the AEZs Lower Midland 1 (LM1), Lower Midland 2 (two areas middle (LM2m) and south (LM2s) split by LM1), Lower Midland 3 (LM3) and Upper Midland 3 (UM3).

For the purposes of the current study, samples from 453 of the 455 IDEAL calves which survived until 51 weeks of age were analyzed, with samples from two calves being unavailable for analysis. There were 181 calves from LM1, 72 calves from LM2m, 70 calves from LM2s, 62 calves from LM3 and 68 from UM3.

### 2.2 Blood samples

Five milliliters of blood was collected at the final routine visit at 51 weeks before the calves left the study. The blood was collected into sterile vacutainer tubes containing EDTA as anticoagulant and stored at −80 °C. DNA was extracted from 250 μl of each blood sample using a blood DNA extraction kit (Invitrogen, Germany) according to the manufacturer's instructions, and eluted in 100 μl of elution buffer.

### 2.3 Reverse line blot (RLB) assay

The RLB hybridization assay was performed as previously described (Gubbels et al., 1999; Bekker et al., 2002; Nijhof et al., 2003, 2005). Briefly, the PCR mixture was prepared using Platinum^®^ Quantitative PCR SuperMix-UDG (Invitrogen, Celtic Molecular Diagnostics, South Africa). Separate PCR master mixes were prepared for amplification of *Theileria* and *Babesia* species (Nijhof et al., 2003), and for amplification of *Ehrlichia* and *Anaplasma* species (Bekker et al., 2002). Reactions were performed in a 25 μl volume with a final concentration of 3 mM MgCl_2_, 20 pmol of each primer, 0.5 U UDG, 200 mM dNTPs, 0.75 U Platinum^®^ Taq DNA polymerase and 100–200 ng of template DNA. A touchdown thermal cycling programme was used as described previously (Nijhof et al., 2005). Mastermix with no DNA template (negative control), and known *A. centrale* and *B. bigemina* DNA samples (positive controls) were included to monitor the occurrence of false positive or false negative results. Probes were covalently linked to a Biodyne^R^ nylon transfer membrane (Pall Corporation, Port Washington, NY, USA). The *Anaplasma, Ehrlichia, Theileria* and *Babesia* genus- and species-specific probes that were included on the membrane are shown in Table 1. PCR products were applied to the membrane and hybridized to the probes using a miniblotter apparatus as described previously (Nijhof et al., 2005). Hybridized PCR products were detected by enhanced chemiluminescence (ECL).

### 2.4 pCS20 quantitative real-time PCR (qPCR)

The pCS20 qPCR was performed as described previously (Steyn et al., 2008), using amplification primers CowF (5′-CAA AAC TAG TAG AAA TTG CAC A-3′) and CowR (5′-TGC ATC TTG TGG TGG TAC-3′), and TaqMan probe Cow™ (5′-6FAM-TCC TCC ATC AAG ATA TAT AGC ACC TAT TA XT-PH-3′). Five microliters of Mastermix pure grade water was used as a negative control and a known *E. ruminantium* positive sample was included to serve as a positive control.

### 2.5 Statistical analysis

The raw prevalence data were used to estimate the population prevalence by adjusting for the stratification by AEZ and the clustering by sublocation used in the design and then by weighting the result by the number of breeding dams in each sublocation using the *R* survey package (Lumley, 2004, 2012). In order to test for clustering of infection in sub-locations, a likelihood ratio test was conducted. The observed distribution of apparent prevalences for each pathogen over the 20 sublocations was compared to the distribution of expected prevalences under the null hypothesis that the prevalences were the same across the study area. A correlation matrix was generated in R using the *rcorr* function and Pearson's method. A *χ*^2^ test for proportions by AEZ was conducted using the *epicalc* package (Chongsuvivatwong, 2008) using the *cc* function.

## 3 Results

### 3.1 Prevalence of haemoparasites in calves at 51 weeks

RLB analysis was undertaken on the final visit (51-week) samples from 453 calves that survived within the IDEAL study period. The analysis showed that 406 (89.6%) calves had at least one detectable haemoparasite, confirming the suspected high prevalence of these parasites in the study region. In all, 15 different species of haemoparasites were detected (Table 2). In addition, 65 and 10 samples produced a signal only with the anaplasma/ehrlichia or the theileria/babesia group-specific probes, respectively, and not with the corresponding species-specific probes. These could not be assigned to a particular species. Table 2 also shows the number of calves which were positive for each of the haemoparasites, together with the haemoparasite prevalences adjusted for the design and weighted by the number of breeding dams per sublocation. The most prevalent species were the theilerial species, *T. mutans* and *T. velifera*, with the two anaplasma species*, Anaplasma* sp*.* Omatjenne and *A. bovis*, also highly prevalent*.* The pathogenic theilerial species, *T. parva*, was present at a much lower prevalence, and only two calves had detectable presence of the pathogenic bacterium *E. ruminantium*. There were very low frequencies of several other haemoparasites, and no samples had detectable levels of *T. buffeli* or *Theileria* sp*.* (buffalo).

We also investigated whether the haemoparasites were detected as single or mixed infections. In this context, mixed refers to infections in which at least two haemoparasite species were detected. Of the 395 calves in which these organisms were identified to the level of species, 344 (87.1%) had mixed infections and 51 (12.9%) carried single infections. Interestingly, several theilerial species were detected only as mixed infections, including *Theileria* sp*.* (sable) and *T. taurotragi*, which were found in 138 and 33 cattle, respectively.

### 3.2 Co-infection analyses

Two co-infection analyses were undertaken on the seven most prevalent infections observed in the IDEAL calves. First, the data were analyzed using correlation coefficients to detect associations between individual species. The results (Table 3) show that the strongest associations were between different theilerial species. *T. velifera* was strongly positively associated with both *Theileria* sp*.* sable and *T. mutans*. There were also strong positive associations between *T. parva* and *T. taurotragi* and between *T. mutans* and *Theileria* sp. sable. The *Anaplasma* parasites showed weaker positive associations with some of the *Theileria* ones, in particular with *T. mutans*, *Theileria* sp. sable and *T. velifera*, but not with each other.

A second co-infection analysis was undertaken to determine how frequently co-infections occurred within and between the two genera. Table 4 shows, for each of the seven most prevalent haemoparasites, the number of samples in which the organism was detected, and the percentage of those samples that were detected as single infections or co-infections with only the anaplasma species (A only), the theilerial species (T only) or both anaplasma and theilerial species (A and T). The results indicate that each haemoparasite was detected as mixed infections in more than 90% of the respective samples. Two species, *Theileria* sp*.* (sable) and *T. taurotragi*, were detected only in the presence of one or more of the other haemoparasites and never as single infections. There were very few co-infections with *Anaplasma* species only, with the percentage of anaplasma co-infections being higher with the theilerial parasites than with each other. Interestingly, both *Anaplasma* sp*.* Omatjenne and *A. bovis* were detected more frequently as co-infections with theilerial parasites only than with (anaplasma and theileria) organisms. In contrast, all of the theilerial parasites were observed mostly as co-infections with anaplasma and other theilerial parasites, rather than theileria alone. The results indicate that the theilerial parasites accommodate co-infections with both theilerial and anaplasma species more readily than do anaplasma organisms, and that the two anaplasma species are much less commonly found as co-infections with each other.

### 3.3 Geographical distribution of the haemoparasites

**Fig. 2:**
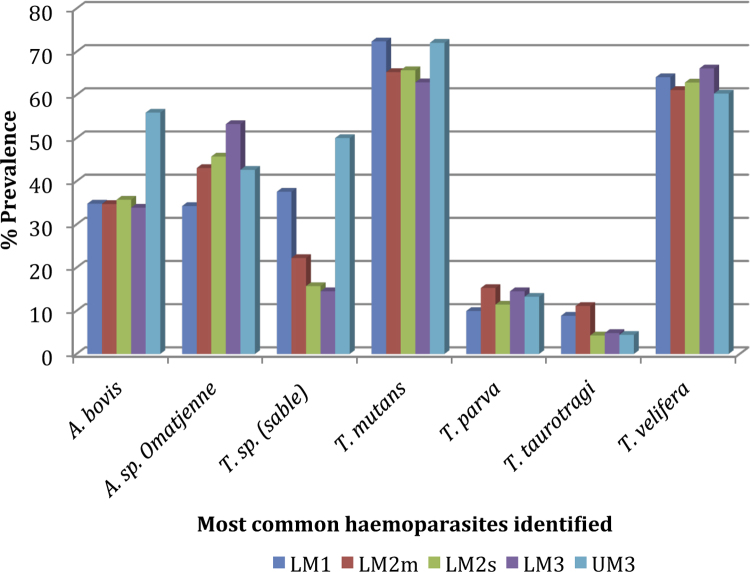
The prevalences of the most common haemoparasites detected by RLB in the different AEZs in the study region.

The distribution of the predominant parasites detected by the RLB across the four AEZs in the study area is shown in Fig. 2. In each AEZ, the basic order of prevalence of these seven parasites was similar. Chi-squared tests on the common species revealed that the only significant associations between haemoparasite prevalance and AEZ (*p* < 0.05) were between the *T.* sp. (sable) and UM3, and between *A. bovis* and UM3, with both parasites being more prevalent in this AEZ (56% and 44%, respectively) than elsewhere.

We also tested for clustering of haemoparasite prevalence by sub-location by conducting a likelihood ratio test. The observed distribution of apparent prevalences for each pathogen over the 20 sublocations was compared to the distribution of expected prevalences under the null hypothesis that the prevalences were the same across the study area. The results, shown in Table 2, indicate a lack of evidence of clustering for most of the parasites, with key exceptions being *Theileria* sp. (sable) and *T. taurotragi*, with weaker but significant clustering being observed for *Anaplasma* sp. Omatjenne and *T. ovis.*

### 3.4 pCS20 qPCR for detection of *E. ruminantium*

The low prevalence of *E. ruminantium* estimated from the RLB results was surprising, given previous reports of the prevalence of this pathogen in Kenya (Ngumi et al., 1997). To confirm the RLB results, all samples were analyzed with the *E. ruminantium*-specific qPCR assay based on the pCS20 gene region. None of the samples returned a positive result with the qPCR assay, including the two samples previously identified as positive by RLB. Despite this discordance, the qPCR assay did confirm the low prevalence of *E. ruminantium* in these samples.

### 3.5 Comparison of RLB and serology results

In a previous study by the group (Kiara et al., 2014), the antibody responses to four of the tick-borne parasites present in the study region (*T. parva*, *T. mutans*, *A. marginale* and *B. bigemina*) were measured by ELISA for each calf at each 5-week time point and analyzed to determine the number of calves that seroconverted to these parasites during the study period. The results revealed that 77% of the calves seroconverted to *T. parva*, 82% to *T. mutans*, 50% to *A. marginale*, and 36% to *B. bigemina* (Kiara et al., 2014). When compared to the adjusted prevalences of these parasites as estimated by RLB (12.1%, 71.6%, 0% and 0.2%, respectively), it is clear that many of the infections with these parasites, apart from *T. mutans*, appear to have been resolved to the extent that the blood stages of the parasites are no longer detectable at 51 weeks.

## 4 Discussion

The study reported here used an RLB assay to determine the prevalences of tick-borne infections in an intensively studied cohort of indigenous calves in a smallholder, mixed crop/livestock farming system in western Kenya, an important farming sytem in eastern Africa. The results are in general agreement with previous studies in the eastern Africa region, albeit in different farming systems containing both indigenous and cross-bred cattle (Oura et al., 2004; Asiimwe et al., 2013). The detection of haemoparasites in cross-sectional analyses is influenced by several factors such as the force of infection experienced by the hosts and the persistence of the parasitaemia which follows infection. Thus a low prevalence may be due to low levels of exposure of the host to the infectious agents, or to resolution of the infection prior to sampling.

In our study, the most common haemoparasites detected were *T. mutans, T. velifera, A. bovis* and *Anaplasma* sp. Omatjenne, the high prevalences of which are consistent with high levels of exposure to the infectious agents and, given the age of the calves, persistent parasitaemias. This was supported for *T. mutans* by the previously available serology data, which showed that 82% of the calf population seroconverted to *T. mutans* during the first year of life, with a mean age at seroconversion of 107 days (Kiara et al., 2014). It is interesting to note that *T. mutans* and *T. velifera* are generally non-pathogenic in cattle (Du Plessis, 1990; Wanduragala and Ristic, 1993).

In contrast, pathogenic haemoparasites were detected at much lower prevalences. Serological data available for three of these organisms (*A. marginale*, *B. bigemina* and *T. parva*) suggest that the low prevalence at 51 weeks is not due to lack of exposure. Rather, the parasitaemias for these organisms generally appear to fall below detectable levels after initial exposure. For *T. parva* for example, 77% of calves seroconverted during the first year of life, with a mean age to seroconversion of 178 days (Kiara et al., 2014), contrasting with the prevalence of 13% at 51 weeks as estmimated by RLB. This difference cannot be ascribed to morbidity as very few of the 34 calves (6.2% of the cohort) that died of *T. parva* infection seroconverted. The difference between seroconversion rate and the prevalence as measured by RLB has been noted previously, with the additional observation that the difference is much less pronounced in crossbred cattle than in indigenous cattle (Oura et al., 2004). The calves in the IDEAL cohort were shown by genomic SNP analysis to be predominantly (83%) of *Bos indicus* origin with about 17% African and European *Bos taurus* introgression (Mbole-Kariuki et al., 2014). Thus, we have confirmed and extended the previous observations by defining more precisely the genotypic composition of the cattle and by including the responses to three other tick-borne organisms. Whether the greater persistence of the parasitaemias observed with the less pathogenic haemoparasites, which would facilitate their transmission, reflects selective evolutionary pressure on the indigenous cattle population remains to be elucidated.

We also observed a very low prevalence of *E. ruminantium*, the causative agent of heartwater, by both RLB and the pCS20 qPCR assay*. E. ruminantium* has previously been isolated in eight districts across Kenya, suggesting that this organism is widely distributed across the country (Ngumi et al., 1997). It is possible that the sequences of the primers and probes used in the RLB assay are not sufficiently similar to those in the corresponding regions in the genomes of the *E. ruminantium* organisms in the study site to allow for detection. The pCS20 sequence, which is the target for the *E. ruminantium*-specific qPCR test, has been well characterized in isolates from South Africa (Van Heerden et al., 2004), but there are limited data on pCS20 sequences from Kenyan *E. ruminantium* strains.

Co-infection of the tick-borne haemoparasites was also examined using the data from the RLB assay. The patterns of occurrence of *T. mutans* and *T. velifera* were similar to each other as were the occurrence of *T. parva* and *T. taurotragi*. This is not unexpected as each pair of parasites is transmitted by similar tick species (*Amblyomma* spp. and *R. appendiculatus*, respectively). A similar pattern was observed in a study on haemoparasites in buffalo in Uganda for *T. mutans* and *T. velifera* (Oura et al., 2011). The weaker but nevertheless statistically significant associations between *T. mutans* and both *T. taurotragi* and *T. parva* may reflect the high prevalence of these parasites, especially the former, and common micro-environmental conditons predisposing to tick exposure. At a more generic level, it was noted that most theilerial infections occurred as mixed infections. This observation, together with the morphological similarity of most theilerial pirpolasms, underscores the difficulty of identifying haemoparasitic infections to species level by microscopy.

The use of the extended version of the RLB assay allowed the detection of organisms of which, to our knowledge, there have been few or no reports in eastern Africa. These included *Theileria* sp*.* (sable), *T. bicornis*, *T. ovis*, *A. phagocytophilum. T. equi* and *E. canis. Theileria* sp. (sable) was originally isolated from a sable antelope (Stoltsz and Dunsterville, 1992), and subsequently identified in clinically healthy cattle in Tanzania (Nijhof et al., 2005) and South Africa (Yusufmia et al., 2010). *T. bicornis* was originally described in South Africa in black rhinoceroses (Nijhof et al., 2003), and more recently in cattle in Uganda (Muhanguzi et al., 2010). *A. phagocytophilum* is the causative agent of tick-borne fever in sheep and cattle in Europe. It has previously been reported in Africa as a possible cause of human granulocytic anasplasmosis the Free State Province, South Africa (Pretorius et al., 1999). The remaining organisms, *T. ovis*, *T. equi* and *E. canis* are parasites of sheep and goats in Africa, horses and dogs, respectively, and are not expected in cattle. Their detection in cattle in our study may be due to aberrant, incidental infections. However, it must also be recognized that there may be novel species of haemoparasites circulating in the study region of western Kenya, with DNA sequences identical or very similar to the probes used in the RLB assay. Such a situation has been recently described for the *Theileria* sp*.* (sable) probe (Brothers et al., 2011). Further, the RLB analysis reported here revealed a signal at the Ehrlichia/Anaplasma group-specific probe and the Theileria/Babesia group-specific probe in 65 and 10 samples, respectively, with no signal at any of the species-specific probes. These results indicate that there are novel *Anaplasma*, *Ehrlichia, Theileria* and *Babesia* spp*.* or variants of known species present in western Kenya which are not identifiable to species level with the current assay format. Thus, confirmation of whether the detection of the unexpected organisms represents true but aberrant infections or whether it is the result of cross-hybridization will require further investigation by sequencing the PCR products derived from the parasites and comparison to known species.

The general lack of association between prevalence of the haemoparasites and AEZ suggests a uniform distribution of the haemoparasites across the study site. The exception was a higher than expected prevalence of *Theileria* sp. (sable) and *A. bovis* in UM3. There was also significant clustering of *Theileria* sp*.* (sable) and *A. bovis* when examined by sub-location. When taken with the AEZ results, the data suggest that these parasites are restricted to sublocations predominantly in UM3. In contrast, there was highly significant sub-location clustering observed for *T. taurotragi*, which was not observed in the AEZ analysis, suggesting that the sublocations in which this parasite is found are not restricted to any particular AEZ. The reasons for these geographical restrictions are not immediately obvious. It should be borne in mind that the occurrence and importance of tick-borne infections is a reflection of complex interactions involving the causative organisms, the vertebrate hosts, the tick vectors, husbandry practices and the environment (Norval et al., 1992). Thus, the reasons for the significant association observed here await further investigation.

## Figures and Tables

**Table 1:** Genus- and species-specific RLB oligonucleotide probes that were used in this study.

Pathogen	Sequence (5′ → 3′)^a^
*Ehrlichia/Anaplasma* group-specific probe (“E/A catch-all”)	GGG GGA AAG ATT TAT CGC TA
*Anaplasma bovis*	GTA GCT TGC TAT GRG AAC A
*Anaplasma centrale*	TCG AAC GGA CCA TAC GC
*Anaplasma marginale*	GAC CGT ATA CGC AGC TTG
*Anaplasma phagocytophilum*	TTG CTA TAA AGA ATA ATT AGT GG
*Anaplasma sp*. Omatjenne	CGG ATT TTT ATC ATA GCT TGC
*Ehrlichia canis*	TCT GGC TAT AGG AAA TTG TTA
*Ehrlichia chaffeensis*	ACC TTT TGG TTA TAA ATA ATT GTT
*Ehrlichia ruminantium*	AGT ATC TGT TAG TGG CAG
*Theileria/Babesia* group-specific probe (“T/B catch-all”)	TAA TGG TTA ATA GGA RCR GTT G
*Babesia* genus-specific probe 1 (“B catch-all 1”)	ATT AGA GTG TTT CAA GCA GAC
*Babesia* genus-specific probe 2 (“B catch-all 2”)	ACT AGA GTG TTT CAA ACA GGC
*Babesia bicornis*	TTG GTA AAT CGC CTT GGT C
*Babesia bigemina*	CGT TTT TTC CCT TTT GTT GG
*Babesia bovis*	CAG GTT TCG CCT GTA TAA TTG AG
*Babesia caballi*	GTG TTT ATC GCA GAC TTT TGT
*Babesia canis*	TGC GTT GAC CGT TTG AC
*Babesia divergens*	ACT RAT GTC GAG ATT GCA C
*Babesia felis*	TTA TGC GTT TTC CGA CTG GC
*Babesia gibsoni* Japan	TAC TTG CCT TGT CTG GTT T
*Babesia gibsoni* USA	CAT CCC TCT GGT TAA TTT G
*Babesia leo*	ATC TTG TTG CTT GCA GCT T
*Babesia major*	TCC GAC TTT GGT TGG TGT
*Babesia microti*	GRC TTG GCA TCW TCT GGA
*Babesia rossi*	CGG TTT GTT GCC TTT GTG
*Babesia vogeli*	AGC GTG TTC GAG TTT GCC
*Theileria* genus-specific probe (“T catch-all”)	ATT AGA GTG CTC AAA GCA GGC
*Theileria annae*	CCG AAC GTA ATT TTA TTG ATT TG
*Theileria annulata*	CCT CTG GGG TCT GTG CA
*Theileria bicornis*	GCG TTG TGG CTT TTT TCT G
*Theileria buffeli*	GGC TTA TTT CGG WTT GAT TTT
*Theileria equi*	TTC GTT GAC TGC GYT TGG
*Theileria lestoquardi*	CTT GTG TCC CTC CGG G
*Theileria mutans*	CTT GCG TCT CCG AAT GTT
*Theileria ovis*	TTT TGC TCC TTT ACG AGT CTT TGC
*Theileria parva*	GGA CGG AGT TCG CTT TG
*Theileria* sp. (buffalo)	CAG ACG GAG TTT ACT TTG T
*Theileria* sp. (kudu)	CTG CAT TGT TTC TTT CCT TTG
*Theileria* sp. (sable)	GCT GCA TTG CCT TTT CTC C
*Theileria taurotragi*	TCT TGG CAC GTG GCT TTT
*Theileria velifera*	CCT ATT CTC CTT TAC GAG T

a The degenerate position R denotes either A or G, W denotes either A or T and Y denotes either C or T.

**Table 2:** The prevalence of haemoparasites in cattle blood samples from western Kenya as determined by the RLB assay.

Species	Total	Raw prevalence	Adjusted prevalence	95% CI	LR test for clustering by SL (*p* value)
*T. mutans*	313	0.691	0.716	0.671–0.760	0.144
*T. velifera*	286	0.631	0.628	0.556–0.700	0.617
*A.* sp*.* Omatjenne	187	0.413	0.427	0.361–0.500	0.025
*A. bovis*	172	0.380	0.399	0.367–0.430	0.078
*T.* sp*.* (sable)	138	0.305	0.327	0.240–0.430	<0.001
*T. parva*	55	0.121	0.129	0.099–0.170	0.325
*T. taurotragi*	33	0.073	0.085	0.056–0.130	0.008
*T. ovis*	14	0.031	0.033	0.018–0.060	0.023
*B. bovis*	10	0.022	0.035	0.013–0.090	0.103
*T. bicornis*	6	0.013	0.013	0.005–0.030	0.419
*E. canis*	2	0.004	0.006	0.002–0.020	0.964
*A. phagocytophilum*	2	0.004	0.004	0.001–0.030	0.909
*E. ruminantium*	2	0.004	0.004	0.001–0.010	0.978
*B. bigemina*	1	0.002	0.003	0.000–0.020	0.997
*T. equi*	1	0.002	0.002	0.000–0.010	0.999
A/E catch-all^a^	65				
T & B catch-alls^a^	10				

a The ‘catch-all’ results are those for which a positive signal was obtained for with the generic probes but not with a species-specific probe.

**Table 3:** Pearson's correlation coefficient for the seven most prevalent species observed (*p* value).

	*A. bovis*	*A.* sp. Omatjenne	*T. mutans*	*T. parva*	*T.* sp. (sable)	*T. taurotragi*	*T. velifera*
*A. bovis*	1.0000	0.000 (1.000)	0.159 (<0.001)	0.030 (0.531)	0.144 (0.002)	0.061 (0.197)	0.098 (0.037)
*A.* sp*.* Omatjenne		1.0000	0.231 (<0.001)	0.073 (0.122)	0.127 (0.007)	0.041 (0.384)	0.176 (<0.001)
*T. mutans*			1.0000	0.117 (0.013)	0.308 (<0.001)	0.096 (0.042)	0.420 (<0.001)
*T. parva*				1.0000	−0.099 (0.035)	0.312 (<0.001)	−0.122 (0.009)
*T.* sp*.* (sable)					1.0000	−0.038 (0.421)	0.506 (<0.001)
*T. taurotragi*						1.0000	−0.156 (<0.001)
*T. velifera*							1.0000

**Table 4:** Co-infection analysis of the seven most prevalent haemoparasites in cattle blood samples from western Kenya, as determined by the RLB assay.

Species	Total (no.)	Single (%)	A only (%)	T only (%)	A and T (%)	Total mixed (%)
*A. sp.* Omatjenne	187	5.9	2.7	56.1	35.3	94.1
*A. bovis*	172	7.6	2.9	51.2	38.4	92.4
*T. mutans*	313	4.2	9.6	22.7	63.6	95.8
*T. velifera*	286	4.5	5.6	24.1	65.7	95.5
*T. sp.* (sable)	138	0	0	21.0	79.0	100
*T. parva*	55	3.6	3.6	27.3	65.5	96.4
*T. taurotragi*	33	0	0	24.2	75.8	100
